# Need of surveillance response systems to combat Ebola outbreaks and other emerging infectious diseases in African countries

**DOI:** 10.1186/2049-9957-3-29

**Published:** 2014-08-05

**Authors:** Ernest Tambo, Emmanuel Chidiebere Ugwu, Jeane Yonkeu Ngogang

**Affiliations:** 1 Sydney Brenner Institute for Molecular Bioscience, Wits 21st Century Institute, Faculty of Health Sciences, University of the Witwatersrand, Johannesburg, South Africa; 2 Center for Sustainable Malaria Control, Department of Biochemistry, Faculty of Natural and Agricultural Sciences, University of Pretoria, Pretoria, South Africa; 3 Faculty of Basic Medical Sciences, Department of Human Biochemistry, Nnamdi Azikiwe University Awka, Nnewi Campus, Nigeria; 4 Faculté des Sciences Biomédicales, Département de Biochimie, Université de Yaoundé, Yaoundé, République du Cameroun; 5 National Institute of Parasitic Diseases, Chinese Center for Disease Control and Prevention, and the WHO Collaborating Centre on Malaria, Schisostomiasis and Filariasis, Shanghai 200025, PR China

**Keywords:** Surveillance response system, Ebola, Outbreak, Emerging infectious diseases, Africa

## Abstract

There is growing concern in Sub-Saharan Africa about the spread of the Ebola virus disease (EVD), formerly known as Ebola haemorrhagic fever, and the public health burden that it ensues. Since 1976, there have been 885,343 suspected and laboratory confirmed cases of EVD and the disease has claimed 2,512 cases and 932 fatality in West Africa. There are certain requirements that must be met when responding to EVD outbreaks and this process could incur certain challenges. For the purposes of this paper, five have been identified: (i) the deficiency in the development and implementation of surveillance response systems against Ebola and others infectious disease outbreaks in Africa; (ii) the lack of education and knowledge resulting in an EVD outbreak triggering panic, anxiety, psychosocial trauma, isolation and dignity impounding, stigmatisation, community ostracism and resistance to associated socio-ecological and public health consequences; (iii) limited financial resources, human technical capacity and weak community and national health system operational plans for prevention and control responses, practices and management; (iv) inadequate leadership and coordination; and (v) the lack of development of new strategies, tools and approaches, such as improved diagnostics and novel therapies including vaccines which can assist in preventing, controlling and containing Ebola outbreaks as well as the spread of the disease. Hence, there is an urgent need to develop and implement an active early warning alert and surveillance response system for outbreak response and control of emerging infectious diseases. Understanding the unending risks of transmission dynamics and resurgence is essential in implementing rapid effective response interventions tailored to specific local settings and contexts.

Therefore, the following actions are recommended: (i) national and regional inter-sectorial and trans-disciplinary surveillance response systems that include early warnings, as well as critical human resources development, must be quickly adopted by allied ministries and organisations in African countries in epidemic and pandemic responses; (ii) harnessing all stakeholders commitment and advocacy in sustained funding, collaboration, communication and networking including community participation to enhance a coordinated responses, as well as tracking and prompt case management to combat challenges; (iii) more research and development in new drug discovery and vaccines; and (iv) understanding the involvement of global health to promote the establishment of public health surveillance response systems with functions of early warning, as well as monitoring and evaluation in upholding research-action programmes and innovative interventions.

## Multilingual abstracts

Please see Additional file 1 for translations of the abstract into the six official working languages of the United Nations.

## The growing public health concern and the burden of Ebola outbreaks

**Figure 1:**
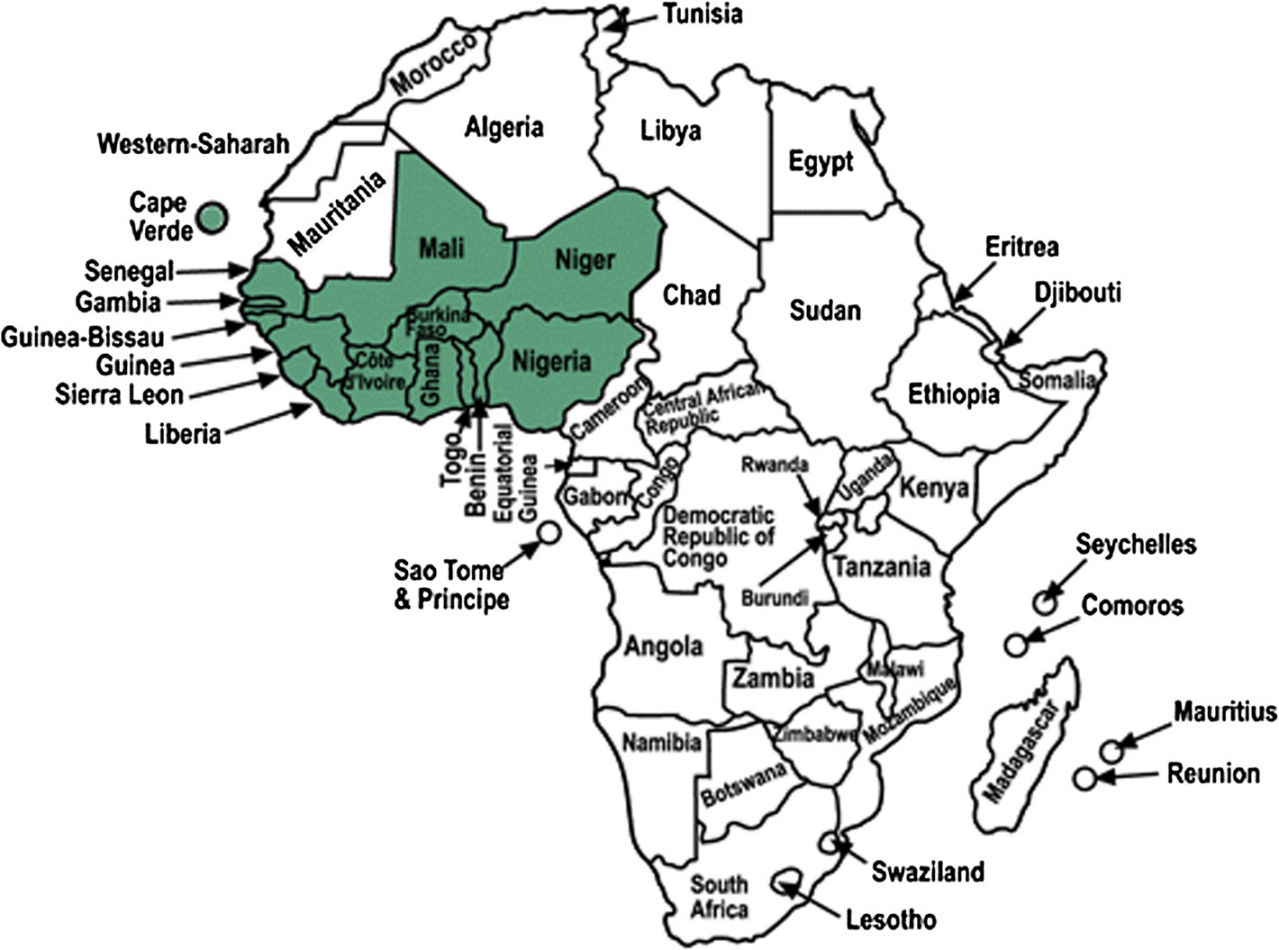
**Western Africa: Economic Community of Western African States (ECOWAS) *Countries: *** **Benin, Burkina Faso, Cape Verde, Cote d’Ivoire, Gambia, Ghana, Guinea, Guinea-Bissau, Liberia, Mali, Niger, Nigeria, Senegal, Sierra Leone and Togo.**

In the absence of an effective drug and vaccine for the dreadful and deadly outbreak caused by the Ebola virus disease (EVD), formerly known as Ebola haemorrhagic fever, there is growing concern for its public health burden in Sub-Saharan Africa. Since 1976, there have been 885,343 suspected and laboratory confirmed cases of EVD, including 1711 cased on the ongoing disease outbreak has claimed 932 lives in West Africa [1]. This part of the world is persistently confronted with this fatal disease which has an incubation period of two to 21 days (averagely 3-13days). Symptoms range from, firstly, fever and fatigue before descending into headaches, vomiting, violent diarrhoea, then multiple organ failure and massive internal bleeding [1,2]. Ebola typically begins in remote places and can be distributed via hospitals/healthcare centers or within the community as it takes several infections before the disease is ascertained. The prevalence, morbidity and case fatality of chronological EVD outbreaks showed the persistent resurgence in different regions in Sub-Saharan Africa (see Figure 1).

Ebola outbreaks have a case fatality rate of 60–90%, yet no specific drug or vaccine is available for people and/or animals hosts. As of 4 August 2014, the cumulative number of cases attributed to EVD in the four countries stands at 1711 including 932 deaths. The distribution and classification of the cases are as follows: Guinea, 495 cases (351 confirmed, 133 probable, and 11 suspected), including 363 deaths; Liberia, 516 cases (143 confirmed, 252 probable, and 121 suspected), including 282 deaths; Sierra Leone, 691 cases (576 confirmed, 49 probable, and 66 suspected), including 286 deaths and Nigeria, 9 cases (0 confirmed, 2 probable, and 7 suspected) including 1 death. Between the 24 and 27 July 2014, a total of 122 new cases (laboratory-confirmed, probable, and suspect cases) of EVD, and 57 deaths, were reported from the four countries as follows: Guinea, 33 new cases and 20 deaths; Liberia, 80 new cases and 27 deaths; Sierra Leone, 8 new cases and 9 deaths; and Nigeria, 1 new case who died [1]. The outbreak is expected to last longer if proper diagnostic tools and rigorous integrated active surveillance response systems are not rapidly established and instituted [3]. Therefore, the following requirements for rapid, scalable and sustainable responses to EVD and other outbreaks across African countries, and globally, have been identified.

## Rapid, scalable and sustainable responses to EVD and other outbreaks of disease

First, the need to urgently recognise and coordinate outbreak action-responses in affected African countries and in cross-border neighbours, as well as collaboration with those that experienced outbreaks in the past, is vital. Overall, Ebola virus socio-ecology systems have shown to be linked by direct and indirect transmission through contact with objects from patients. For example, the blood or secretions of an infected person or objects that have been contaminated with infected secretions can reach humans from a variety of hosts/sources: naïve infected populations, infected wildlife, fruit and vegetable bats and the handling of infected fruit bats, monkeys, chimpanzees, gorillas, forest antelopes and porcupines are all possible natural hosts (whether ill or dead or found in the jungle or rainforest) [1,2]. Thus, tracking, mapping, reporting and documenting veterinary public health zoonosis surveillance responses, the behaviour and medical history of butchers and poachers, as well as agro-livestock business trading are imperative to be able to establish integrated community-based and national comprehensive early warning and outbreak surveillance response systems.

Second, understanding the unending transmission dynamics and resurgence is essential to actively identify and map transmission foci and local micro-epidemiological situations, which can lead to implementing prompt, effective response interventions tailored to specific local settings. Hence, active early warnings approach under the framework of a surveillance response system both for veterinary and human public health should be established and implemented. This system could include, for example, a ban on bush meat consumption, a ban on public places and markets, a reinforcement of safety and inspection regulations on food and fruit eating bats and examining bat migration as well as other animal-household drivers and risk factors [3,4].

Third, instituting electronic-based reporting systems based on advances in information and communication technologies (ICTs) is crucial as already about 40% of the West African affected populations use mobile phones (*m*health or *e*health). Building a local network (e.g. WhatsApp) or crowdsourcing data for targeted active responses, as well as the implementation of a geographical information system (GIS), are necessary spatial-temporal mapping and decision-making support systems to contain Ebola outbreaks. Lessons learnt from other outbreaks including cholera, H7N9 and H1N1 avian influenza, severe acute respiratory syndrome (SARS), Lassa fever, the Middle East respiratory syndrome (MERS), dengue pandemic and the human-animal with environmental-climate interface in Africa and elsewhere can assist in setting benchmarks for monitoring epicentre/focal early warning alert, incidence and prevalence as well as effective surveillance response interventions measures [1,5]. Meanwhile, modelling factors and trends in different changing transmission scenarios could also yield better tactics, as well as strategic evidence not only for policy support, but also for direction, planning and implementation of national and regional early alert and surveillance response systems to control and prevent sentinel sites [4]. Following from the aforementioned three challenges and requirements to respond to EVD outbreaks in Africa, the following actions are recommended to combat EVD as well as other emerging infectious diseases.

## Mapping the unprecedented geographical migration of EVD across Africa

Ebola was first reported in 1976 in Sudan and Congo and named after the river where it was identified. It was later reported in Gabon, Central Africa. The genus *Ebolavirus* is one of three members of the *Filoviridae* family (*filovirus*), along with the genus *Marburgvirus* and the genus *Cuevaviru*s, and comprises five distinct species, of which *Zaire ebolavirus, Sudan ebolavirus* and *Bundibugyo ebolavirus* are mostly associated with the major EVD outbreaks in Africa. *Reston ebolavirus* and *Taï Forest ebolavirus* are mostly reported in the Asia-Pacific region, especially Philippines and the People’s Republic of China, but no symptoms or deaths in humans from these have been reported to date [2]. The disease outbreak has persisted over the years across Central, East and Southern Africa. On March 21, 2014, the first case of an outbreak was identified in Guinea, West Africa, witnessing a total of 460 cases with 339 fatalities, as it spread beyond the remote rural areas to the capital city of Conakry. Soon after the outbreak was identified, it appeared across the border in the small nation of Liberia on March 30, 2014. This country was the least hit with 339 cases and 156 fatalities thus far. Later, it was also identified in Sierra Leone in late May 2014, just as it appeared the outbreaks in Guinea and Liberia were winding down. It has since spread to at least two Sierra Leone districts with 533 cases claiming at least 233 lives, including 68 deaths of 85 new cases within just four days. Similarly, between 21 and 23 July 2014, 96 new cases and 7 deaths were reported from Liberia and Sierra Leone. In Guinea, 12 new cases and 5 deaths were reported during the same period and one death in Nigeria [1] (see Table 1).

**Table 1:** Geographical and spatiotemporal epidemiology of Ebola virus disease outbreaks across Africa from 1976 to 2014

***Countries***	***Species/year***	***Total cases***	***Total deaths***	***Diagnosis***	***Existing interventions***	***Challenges and prospects***
**South Sudan**	*Sudan ebolavirus,* 1976	284	151		– Balancing infusion of fluids/electrolytes	– Early stage diagnosis
	*Sudan ebolavirus,* 1979	34	22	– Syndromic or retrospective assessment	– Maintaining oxygen status and blood pressure	– Effective mass screening tools
	*Sudan ebolavirus,* 2004	17	7			
						– Novel drugs in management
**Congo**	*Zaire ebolavirus*, 2001–02	59	44			
	*Zaire ebolavirus*, 2003	178	157		– Management of complicated cases	– Primary prevention measures such as education, capacity building, training and advocacy
	*Zaire ebolavirus*, 2005	12	10			
**Gabon**	*Zaire ebolavirus*, 1994	52	31			
				–Antibodies ELISA IgM and IgG assay	– Community mobilisation, participation and health education	
	*Zaire ebolavirus*, 1996	91	61			
	*Zaire ebolavirus*, 2001–02	65	53			– Human-animal migration
**Ivory Coast**	*Taï Forest ebolavirus,* 1994	1	0			
**DRC**	*Zaire ebolavirus*, 1976	318	280			
	*Zaire ebolavirus*, 1977	1	1		– Routine cleaning and disinfection of host farms	– Resettlements and wars
	*Zaire ebolavirus*, 1995	315	250			
	*Zaire ebolavirus*, 2005	32	15			
				– Immunological testing		
	*Zaire ebolavirus*, 2007	264	187			– Poaching
					– Close supervision of burial or incineration of carcasses	– Deforestation and forest degradation
				Serum neutralisation test		– Climate change and global warming
					– Personal protective measures	– Infrastructure factors
				– Molecular assays such as qPCR – Electron microscopy		– Animal-environmental factors
	*Zaire ebolavirus*, 2008	32	14			– Trans-trading, mining and urbanisation
	*Zaire ebolavirus*, 2012	36	13			
**South Africa**	*Zaire ebolavirus,* 1996	1	1			– Poverty cycle
**Uganda**	*Zaire ebolavirus,* 2000	425	224			– Health education
	*B. ebolavirus,* 2007	149	37	– Virus isolation and susceptibility testing		– Community participation and empowerment and bioinformatics
	*Zaire ebolavirus,* 2011	1	1			
	*Sudan ebolavirus,* 2012	31	21			
**Guinea**	*Zaire ebolavirus,* 2014	495	363			
**Sierra Leone**	*Zaire ebolavirus,* 2014	691	281			
**Liberia**	*Zaire ebolavirus,* 2014	516	286			
**Nigeria**	*Zaire ebolavirus,* 2014	9	1			

DRC: Democratic Republic of Congo, Congo: Republic of Congo, ICT: Information and Communication Technology.

## Understanding African cultural and customs practices and how they affect psychosocial-behavioural attitudes towards Ebola outbreaks

**Figure 2:**
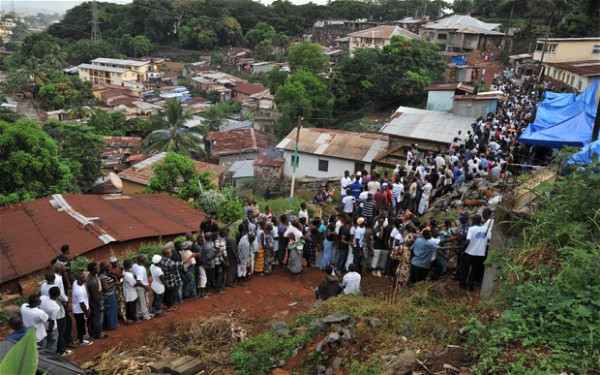
Ebola virus blood testing by government health workers in the Kenema district, Sierra Leone, June 25, 2014.

Socio-demographically, Western Africa, the Economic Community of Western African States (ECOWAS) is made up of 15 countries with a population of about 340 million and a population density of 49.2 persons/km^2^ and 127.5 persons/square mile. Nigeria accounts for half of the population and half of the regional aggregate. The ECOWAS has a total area of 5,112,903 km^2^. The total GDP (PPP) is US$ 752,983 billion and US$ 2,500 per capita, making ECOWAS the single largest economic and trading union in Africa and one of the pillars of the African economic community according to the World Bank, 2013. Each country consists broadly of two distinct zones: a sahelian zone (North), largely landlocked, and a more humid, forested coastal zone (South), with the literacy rate varying from 41-62% (see Figure 1).Similar to the conception and spread of the HIV/AIDS pandemic in Africa, one of the main obstacles in reducing the distribution of Ebola has been the widespread ignorance, lack of knowledge and potential panic over EVD, considered to be a ‘Satanic or bewitched’ disease, leading to trepidation, isolation, dignity impounding, stigmatisation and ostracism from associated socio-ecological and public health consequences. Recently, local residents of the Sadialu village in Sierra Leone were sheltering those infected with Ebola, refusing to go to or escaping from hospitalisation referred as “death sentence”, and hiding from the local health centre due to circulating beliefs, myths and rumours that the interventions being administered to patients were actually causing the disease (see Figure 2).

Such consequences and misconceptions are proscribed in the International Health Regulations (IHR), human rights laws, as well as the Helsinki and the WHO declarations. These detail the challenges and opportunities that Ebola and other infectious diseases are currently facing, including neglected tropical disease (NTD) prevention, control and management, as well as health system coverage and service delivery bottlenecks. Harnessing all stakeholders’ collaboration, communication and networking, including communities, is essential for improving and nurturing community participation, informal and formal health education, ownership and empowerment of the programmes, and patient independence and dignity, as well as ensuring human rights for all ages at all levels in order develop a productive and sustainable African continent and achieve the Millennium Development Goals (MDGs) and global health.

Hence, accelerating the response through provision of minimum essential information on risk communication for behavioural impact, developmental communication and health promotion/education personnel and community, working in multidisciplinary to respond to the disease outbreak, will be very useful for adequate and appropriate national staff and other national resources to the field operational epidemiologists, clinicians, and public health officers in fostering positive behavioural changes while respecting cultural practices, and impact on local contexts and outbreak dynamics, prevention and control interventions and scaling up outbreak containment measures, especially effective contact tracing.

## Developing surveillance response systems against Ebola and other infectious disease outbreaks

Unfortunately, several countries in Africa, as well as governmental and research institutions, are inadequately equipped in diagnostics, tracking, active reporting, prompt healthcare delivery, and accessible and affordable treatment to combat the Ebola infection and other emerging infectious diseases. The development of new tools, strategies and approaches, such as improved diagnostics and novel therapies including vaccines, is needed to prevent, control and contain Ebola as well as SARS, bird flu, Lassa fever, dengue and MERS outbreaks. Hence, the urgent need to develop and implement early warning alert and active surveillance response systems for emerging infectious diseases and the control and elimination of NTDs, as well as early warning and emergency systems, cannot be overemphasised. The prerequisites for fighting and containing the transmission and saving lives include concerted actions to empower communities through mobilisation, communication and participation; formal and informal education; and training of community and environmental health professionals. Timely and effective reporting, documentation and communication of incidence and prevalence by all stakeholders including the health ministries, international and local NGOs, UN agencies, religious leaders, WHO regional and Partners (CDC, MSF, UNICEF, IFRC) continue to work together through the Sub-regional Ebola Outbreak Coordination Center (SEOCC), global health institutions and other stakeholders are paramount in early containment response [4]. WHO does not recommend any travel or trade restrictions is applied to Guinea, Liberia, Sierra Leone or Nigeria, based on the current information available. Moreover, people who don’t have the knowledge should be educated on how to protect themselves. Also important is the prompt quarantining of the sick and the dead in line with the African customs and burial traditional, cultural myths and practices, as it is believed in such cultures that corpses are still contagious and customary transmits the disease.

## Implementing new surveillance tools and strategies to combat Ebola outbreaks and other infectious diseases

**Figure 3:**
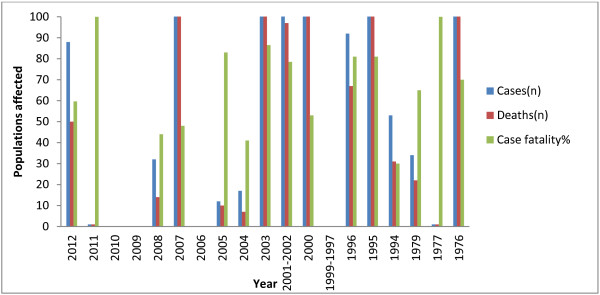
Chronology of Ebola virus disease outbreaks in affected African countries from 1976 to July 2014.

There is an urgent necessity to strengthen the primary healthcare system, and develop more sensitive serological and molecular diagnostic tools, as well as innovative methods and approaches to assess vulnerability in agreement with current practices (see Figure 3). This requires further research and development (R&D), capacity building based on international best practices for containing public outbreaks, the drafting of standard processes and operating procedures, biorisk management as samples from patients and animals are an extreme biohazard risk, thorough adherence to the WHO global alert and response operations, and outbreak communication guidelines. Moreover, maximizing the advances in genomic, biotechnological and communication technologies provides efficient and improved surveillance tools for early warning system prognostic, monitoring and evaluation control and prevention of outbreaks; these should be based on preventing the source as ascribed in the ‘One World-One Health’ standpoint [2,6]. In addition, intensive efficacy and pharmacovigilance assessment of these interventions including diagnostics, drugs and vaccines against Ebola and other emerging infectious diseases including NTDs must be carefully re-evaluated, and the cut-offs determined and monitored over time, in addition to enhance cross-border collaboration and strengthen effective coordination across African government and populations [2-4].

Real-time active surveillance response systems, research priorities and innovative mechanisms for outbreaks include the development of tools targeting early active diagnosis especially at the onset and during the low level of transmission; tracking and mapping; monitoring human and host population migration; forecasting outbreaks based on risk factors; assessment of indicators and minimal essential datasets to guide evidence decision making; strategic planning and effective control; and prevention programmes and response packages tailored to local settings [4,5]. Experiences and lessons learnt from outbreaks in developed nations could be shared with limited-resource countries so that they can to establish early warning and surveillance response systems [4]. Irrefutably, national and regional inter-sectorial and trans-disciplinary approaches must be adopted and related to ministries and organisations in order to build innovative early warning system surveillance response systems through fostering capacity building and training on outbreaks and emerging infectious disease prevention, control and elimination. It is also imperative to understand global health involvement and governance, establish monitoring and evaluation (M&E) of research for action programmes, as well as increase funding to support efforts of existing and new consortiums and research projects in Africa. Furthermore, there is need to analyse the socio-economic and cultural factors, the status of prevailing health systems, and the risk factors and determinants of the emergence and spread of outbreaks in Africa. Reliable and well-organised monitoring, the establishment of GISs and appraisals of cost-effectiveness in an integrated national health system (with a care management approach) will eventually improve evidence information for policy-makers [4]. In turn, they can make decisions and guide implementers of health programmes to achieve beneficial and innovative sustainable global public health interventions, quality healthcare outcomes and economic prosperity.

## Conclusion

Consolidating and harmonizing the technical support at local, country, regional, and international level is required in mobilizing the international community in support of national efforts in epidemic and pandemic prevention and control. Hence, developing, scaling up and strengthening all aspects of the outbreak surveillance response system including contact tracking, public information and community mobilization, case management and infection prevention and control, and effective coordination.

## Competing interests

The authors declare that they have no competing interests.

## Authors’ contributions

ET conceived, collected and analysed the data, and drafted the manuscript. ET, ECU and JYN provided additional information. All authors read and approved the final manuscript.

## Supplementary Material

Additional file 1 Multilingual abstracts in the six official working languages of the United Nations. Click here for file
