# Supplementary material for: Need of surveillance response systems to combat Ebola outbreaks and other emerging infectious diseases in African countries
Source: Infect Dis Poverty. 2014 Aug 5;3:29. doi: 10.1186/2049-9957-3-29 (PMC4130433; doi:10.1186/2049-9957-3-29)
Supplement: Additional file 1 — Multilingual abstracts in the six official working languages of the United Nations. [file 2049-9957-3-29-S1.pdf]

Translation of the abstract into the six official working languages of the United Nations

الحاجة إلى نظام استجابي رقابي لمكافحة تفشي مرض الإيبولا وغيرها من الأمراض المعدية الناشئة في البلدان الأفريقية.

إرنست تامبو، ايمانويل تشيديبير أوجو، جين يونكو نجوجانج

ملخص

مرض فيروس إيبولا (EVD) والمعروف سابقاً بحمى الإيبولا النزيفية. هناك قلق متزايد في أفريقيا - جنوب الصحراء الكبرى بشأن تفشي مرض الإيبولا والعبء الموضوع على الصحة العامة. هذا المرض الذي تسبب في 884,764 حالة بين مشتبه فيها ومؤكدة مختبرياً وأودى بحياة 603 من الشعوب الأفريقية منذ 1976.

تم تحديد خمسة تحديات واحتياجات للاستجابة لتفشي مرض الإيبولا: (1) نقص في تنمية وتنفيذ نظام استجابي رقابي ضد مرض الإيبولا وغيرها من الأمراض المعدية في أفريقية. (2) ضعف التعليم والمعرفة والوعي للاستجابة لمرض الإيبولا مما يبعث بالذعر والقلق والصدمة النفسية والعزلة وامتهان الكرامة والوصمة والنبذ المجتمعي والمقاومة وارتباط ذلك بآثار بيئية مجتمعية وأثار مرتبطة بالصحة العامة. (3) موارد مالية وقدرات بشرية تقنية محدودة، ضعف الخطط العملية لنظام الصحة الوطنية والمجتمعية للوقاية وظبط الاستجابة والممارسات والإدارة . (4) عدم كفائة القيادة والتنسيق. (5) نقص في تنمية استراتيجيات وآليات وأساليب جديدة مثل تحسين التشخيص وإيجاد علاجات حديثة بما في ذلك اللقاح الذي بإمكانه المساعدة في الوقاية والسيطرة واحتواء تفشي مرض الإيبولا وانتشاره. ومن ثما فهناك احتياج لتنمية وتفغيل نظام استجابي رقابي نشط لمواجهة تفشي المرض وللسيطرة على الأمراض المعدية الناشئة. إن فهم المخاطر التي لا تنتهي من ديناميكية انتقال العدوى وعودة ظهورها، هي من الأمور الهامة لتفعيل تدخلات استجابية فعّالة مصممة خصيصاً للظرف والسياق المحلي المحدد.

علاوة علي ذلك، ينصح بالإجراءات التالية: (1) نظام استجابي رقابي على المستوى الوطني وبين القطاعات والتخصصات المحلية، تأخذ نهج الإنذار المبكر، بالإضافة إلى تنمية الموارد البشرية الحرجة. كل ذلك يجب أن تتبناه الوزارات والمنظمات المتحالفة في الدول الأفريقية في الاستجابة للأمراض الوبائية. (2) تسخير كل أصحاب المصلحة والتزامهم في التمويل المستدام والتعاون والتواصل والاتصال مع المشاركة المجتمعية في تعزيز الاستجابة المنسقة وتتبع وادارة الحالات لمكافحة تلك التحديات. (3) المزيد من الأبحاث والتنمية لاكتشاف أدوية ولقاحات جديدة. (4) ادراك أن مشاركة الصحة العالمية سيعزز من تأسيس نظم استجابية رقابية للصحة العامة والتي ستعمل على الانذار المبكر ورصد وتقييم برامج البحوث العملية والتدخلات المبتكرة.

Translated from English version into Arabic by T. Catherine Hanna, through

TRANSLATORS WITHOUT BORDERS

非洲国家应对埃博拉病毒病爆发和其他新发传染病的监测响应体系需求

Ernest Tambo, Emmanuel Chidiebere Ugwu, Jeane Yonkeu Ngogang

摘要

埃博拉病毒病（Ebola virus disease，EVD），原名埃博拉出血热，该病的爆发及其公共卫生负担在撒哈拉以南非洲地区越来越引起关注，自 1976 年起，该病已致非洲 884,764 例疑似和确诊病例，报告死亡人数为 603 人。响应该病爆发面临有以下五个挑战和需求：1.非洲应对埃博拉病毒病和其他传染病爆发的监测响应体系发展与实施的不足；2.缺乏对埃博拉病毒病的教育与知识，因缺乏相关知识引起的恐慌、焦虑、心理创伤、受侮辱、被群体排斥等相关的社会生态学和公共卫生后果；3.在防治应答、实践和管理方面有限的经济资源和人员技术能力，以及薄弱的社区和国家卫生体系工作计划；4.领导力和协调能力不足；5.缺少发展新策略、新工具和方法，例如一些更好的诊断方法和包括疫苗在内新的治疗手段，而这些均有助于预防、控制和应对埃博拉病毒病爆发与扩散。因此，迫切需要发展和实施新发传染病爆发响应和控制的主动监测响应体系。并且了解其传播动力学和复燃的风险是实施适合特定情况下快速有效的应答措施必不可少的。

此外，推荐以下工作方案：1.非洲国家间快速联合不同部委和组织采用包括早期预警和关键性人力资源开发在内的全国性和区域性部门间和跨学科监测响应体系；2.利用所有利益攸关方在持续资助、合作、信息和网络建设方面的承诺，包括克服上述挑战的协同应答提高、追踪病例和及时的病例管理等；3.更多的新药和疫苗研发；4.了解参与全球卫生对促进公共卫生监测响应体系建立的意义，该体系具有在支撑研究-行动方案和创新性干预措施方面的早期预警、督导和评估功能。

Translated from English version into Chinese by Yin Jian-hai, through

CHINESE CENTER FOR DISEASE CONTROL AND PREVENTION
NATIONAL INSTITUTE FOR PARASITIC DISEASES

De la nécessité d'un système de surveillance et de réponse dans la lutte contre les épidémies du virus Ebola et autres maladies infectieuses émergentes dans les pays africains

Ernest Tambo, Emmanuel Chidiebere Ugwu, Jeane Yonkeu Ngogang

Résumé

La maladie à virus Ebola (autrefois appelée aussi fièvre hémorragique à virus Ebola), est de plus en plus préoccupante en Afrique subsaharienne, où sa propagation épidémique crée un poids important pour la santé publique avec 884 cas suspects, 764 confirmés en laboratoire et 603 décès dans les populations africaines depuis 1976. Face aux épidémies du virus Ebola, cinq difficultés ont été identifiées : (i) les carences de développement et de déploiement d'un système de surveillance et de réponse aux flambées du virus Ebola et d'autres maladies infectieuses en Afrique, (ii) le manque d'éducation et de connaissances sur les émidémies de MVE, suscitant panique, inquiétude, traumatisme psychosocial, isolement et perte de dignité, stigmatisation, ostracisme et résistance communautaire, avec les conséquences socio-écologiques et sanitaires qui en découlent, (iii) les limites des ressources financières, des moyens techniques humains et des plans d'action des systèmes de santé locaux et nationaux pour la prévention et la lutte contre la maladie, des pratiques et des méthodes de gestion, (iv) une direction et une coordination insuffisantes et (v) l'absence de nouvelles stratégies, approches et outils, par exemple d'instruments de diagnostic améliorés et de moyens thérapeutiques (y compris les vaccins) pour prévenir les flambées du virus Ebola, les contrôler et juguler leur propagation. Il est donc urgent de développer et de déployer un système de surveillance et de réponse active pour réagir aux épidémies et lutter contre les maladies infectieuses émergentes. La compréhension des risques infinis de la dynamique de la transmission et de la résurgence est indispensable pour déployer des interventions efficaces, adaptées au contexte et aux conditions locales.

Les actions suivantes sont également préconisées : (i) un système de surveillance et de réponse intersectoriel et transdisciplinaire, national et régional, avec alerte précoce, doté des moyens humains indispensables, doit être adopté rapidement par les ministères et organismes alliés dans les pays africains, en réponse aux épidémies et aux pandémies ; (ii) toutes les parties prenantes doivent s'engager durablement dans le financement, la collaboration, la communication et la création de réseaux, avec la

participation des communautés, afin de renforcer et de coordonner la réponse, de pérenniser et d'encourager la gestion des cas pour résoudre ces difficultés ; (iii) la recherche sur les nouveaux médicaments et les vaccins doit être intensifiée ; (iv) les structures de santé mondiales doivent être impliquées afin de favoriser la création de systèmes de surveillance et de réponse sanitaires assurant aussi bien les alertes avancées que la surveillance et l'évaluation en vue de mettre en place des programmes de recherche et d'action et des interventions innovantes.

Translated from English version into French by Suzanne Assenat, through

TRANSLATORS
WITHOUT BORDERS

Необходимость системы ответного реагирования в борьбе со вспышками Эболы и другими новыми инфекционными заболеваниями в Африканских странах.

Эрнест Тамбо, Эммануэль Кидьебере Угву, Жан Йонкё Нгоганг

Реферат

Болезнь, вызванная вирусом Эбола (известная ранее как геморрагическая лихорадка Эбола) вызывает растущую озабоченность в странах Африки, расположенных к югу от Сахары, в связи с распространением вспышек этого заболевания и вызванной ими нагрузкой на национальные системы здравоохранения: с 1976 года они явились причиной 884764 подозреваемых и лабораторно подтвержденных смертей и еще 603 заявленных смертей африканского населения. Определены пять основных проблем в организации ответных действий на вспышки вируса Эбола: (i) недостатки в разработке и реализации систем ответного реагирования на вспышки Эболы и других инфекционных заболеваний в Африке, (ii) недостаточная просвещенность и осведомленность о вирусе Эбола, вызывающие панику, тревожность, психосоциальный травматизм, изоляцию и унижение достоинства, социально-психологическую дискриминацию, остракизм и сопротивление, сопровождаемые социально-экологическими последствиями и влиянием на здоровье населения, (iii) ограниченные финансовые ресурсы, уровень технического развития, недостаточно развитые планы действия местных сообществ и системы здравоохранения по профилактике и управлению и методам реагирования, (iv) не отвечающие требованиям руководство и координация (v) нехватка новых стратегий, инструментов и подходов, таких как более совершенные диагностические средства и новые методы терапии, включая вакцинацию, которые могут помочь в предотвращении, контроле и сдерживании вспышек и распространения болезни Эбола. Отсюда следует острая необходимость в создании и реализации активной системы ответного реагирования в борьбе со вспышками Эболы и других новых инфекционных заболеваний. Понимание бесчисленных рисков динамики переноса имеет первостепенное значение для принятия своевременных и эффективных мер, отвечающих специфическим местным условиям и контексту.

Кроме того, рекомендуется проведение следующих мероприятий: (i) родственные министерства и организации африканских стран должны создать национальные и региональные межотраслевые и трансдисциплинарные системы ответного реагирования, основанные на подходе раннего предупреждения и подготовки необходимых человеческих ресурсов для борьбы с эпидемиями и пандемиями, (ii) объединение усилий всех заинтересованных сторон в сфере долгосрочного финансирования, сотрудничества, создания сети контактов, включая участие общественности в укреплении координированного реагирования, отслеживания и управления в решении таких проблем, (iii) расширение исследовательской работы по созданию новых медикаментов и вакцин, (iv) понимание значения всемирной охраны здоровья в создании систем по надзору в сфере здравоохранения с функциями раннего предупреждения, а также мониторинга и оценки программ исследований и инновационных мероприятий.

Translated from English version into Russian by Alena Hrybouskaya, through

TRANSLATORS
WITHOUT BORDERS

Necesidad de un sistema de vigilancia de la respuesta para combatir los brotes de Ébola y otras enfermedades infecciosas emergentes en los países africanos

Ernest Tambo, Emmanuel Chidiebere Ugwu, Jeane Yonkeu Ngogang

Resumen

La enfermedad del virus del Ébola (EVD, por su sigla en inglés), anteriormente conocida como fiebre hemorrágica del Ébola, es motivo de creciente preocupación en el África subsahariana por la propagación de los brotes de esta enfermedad y por la carga de la salud pública que han sido la causa de 884.764 muertes sospechadas y confirmadas por laboratorio y que cobró 603 vidas de las poblaciones africanas desde 1976. Se identifican cinco desafíos y necesidades de respuesta a los brotes de EVD: (i) deficiencia en el desarrollo e implementación del sistema de vigilancia de la respuesta para combatir los brotes de Ébola y de otras enfermedades infecciosas en África (ii) falta de educación y conocimiento de la respuesta a los brotes de EVD que provocan pánico, ansiedad, traumatismo psicológico, aislamiento y privación de la dignidad, estigmatización, ostracismo y resistencia de la comunidad, con las consecuencias socio ecológicas y de salud pública asociadas (iii) recursos financieros y capacidad técnica humana limitados, y planes operativos comunitarios y del sistema nacional de salud débiles en las respuestas, prácticas y gestión de la prevención y del control (iv) liderazgo y coordinación inadecuadas y (v) falta de desarrollo de nuevas estrategias, herramientas y enfoques, como la mejora de los diagnósticos y de nuevos tratamientos, incluyendo la vacunación que puede ayudar a prevenir, controlar y contener los brotes y la propagación del Ébola. De ahí, la urgente necesidad de desarrollar e implementar un sistema de vigilancia activa de la respuesta a los brotes y del control de las enfermedades infecciosas emergentes.

Comprender los riesgos interminables de la dinámica de la transmisión y del resurgimiento es esencial para la implementación de intervenciones de respuesta rápidas y eficaces adaptadas a las condiciones locales específicas y al contexto.

Además, se recomiendan las siguientes acciones: (i) la rápida adopción por los ministerios y organizaciones aliadas entre los países africanos de un sistema nacional y regional de vigilancia de respuesta intersectorial y transdisciplinario que incluya el enfoque de alerta temprana, así como el desarrollo fundamental de los recursos humanos en las respuestas epidémicas y pandémicas (ii) aprovechar el compromiso de todos los interesados para lograr el financiamiento sostenido, la colaboración, la

comunicación y la creación de redes, incluyendo la participación de la comunidad para mejorar la respuesta coordinada, el seguimiento y la pronta gestión de los casos para luchar contra esos desafíos (iii) más investigación y desarrollo para descubrir nuevos medicamentos y vacunas, (iv) entender la participación de la salud mundial para promover el establecimiento de sistemas de vigilancia de la respuesta de salud pública con funciones de alerta temprana, así como el seguimiento y la evaluación en la defensa de los programas de investigación y acción e intervenciones innovadoras.

Translated from English version into Spanish by Susana Rosselli, through

TRANSLATORS
WITHOUT BORDERS
